# Study of the Impact of Coals and Claystones on Wear-Resistant Steels

**DOI:** 10.3390/ma16062136

**Published:** 2023-03-07

**Authors:** Andrzej N. Wieczorek, Iwona Jonczy, Krzysztof Filipowicz, Mariusz Kuczaj, Arkadiusz Pawlikowski, Dariusz Łukowiec, Marcin Staszuk, Anna Gerle

**Affiliations:** 1 Faculty of Mining, Safety Engineering and Industrial Automation, Silesian University of Technology, Akademicka 2, 44-100 Gliwice, Poland; 2 Faculty of Mechanical Engineering, Silesian University of Technology, Konarskiego 18A, 44-100 Gliwice, Poland; 3 Łukasiewicz Research Network—Institute of Ceramics and Building Materials, Refractory Materials Division in Gliwice, Toszecka 99, 44-100 Gliwice, Poland

**Keywords:** wear, coal, claystone, tribology, wear-resistant steels

## Abstract

This paper discusses the impact of coal abrasive materials of varied petrographic composition and claystones containing admixtures of coal matter on the surface wear of wear-resistant martensitic steels. Wear tests were conducted at a test stand for three petrographic varieties of hard coal: vitrinite, clarinite, and durinite, and five samples of claystone. These tests revealed no significant effect of the type of coal abrasive used on the value of mass loss from the surface of the wear-resistant steel samples. The reason behind the foregoing is the observed tendency of coal abrasives, irrespective of their petrographic variety, to penetrate surface irregularities, especially those attributable to previous surface treatment of the samples and the impact of wear products. The dominant forms of surface damage were surface fatigue chipping and scratches caused by the particles which detached themselves from the surface of the steel samples, as observed for all the analysed coal variants. On the surfaces of the samples seasoned in the presence of claystones, highly varied forms of damage were observed: microcutting, scaly surface cracks, delamination, and deep cracks. In these cases, it was possible that the abrasive grains had been pressed into the steel surface irregularities, but no layered forms of the pressed-in abrasive material were observed to have developed. The paper also presents a model for the formation of coal films and discusses their possible effect on wear minimisation.

## 1. Introduction

Wear is caused by friction processes, most of which involve changes to the mass, volume, or physical properties of the superficial layers in the interfaces between mating surfaces. The intensity of the wear process depends on the resistance of the materials forming the friction node to a given type of failure [1,2]. The form of wear causing the greatest economic losses during the operation of working machines is abrasive wear. Surfaces of the components of the machinery used in the mining industry for extraction of minerals and energy resources [3,4] are particularly exposed to this form of wear. 

Abrasive wear causing damage to the superficial layer of steel machine elements occurs when there are abrasive particles or wear products in the friction areas of mating elements. The material loss in the superficial layer may take place as a result of such processes as microridging, microscratching, microfatigue, and microcracking [5]. In the case of abrasives containing grains characterised by high hardness, damage occurs as a result of microscratching or microridging. For softer rocks, other wear mechanisms may occur, such as surface fatigue, caused by the cyclic interaction of the abrasive grains along the wear surface. As a result of this interaction, elastic, elastic-plastic, or plastic deformations occur in the superficial layer, resulting in the development of cracks by way of shallow chipping (delamination). 

In the abrasive wear processes of the three body type [6,7], more than one destructive mechanism may be involved. This is due to the fact that, in real-life conditions, the abrasives penetrating into the mating areas of machine elements are most often mixtures of minerals characterised by different hardnesses and, consequently, they have different impacts on the surfaces of these elements. 

One of the most economically important minerals is hard coal. Coal seams are very often accompanied by claystones containing admixtures of coal. In mining operations, machine surfaces usually sustain intensive damage through abrasive wear [8,9,10]. An example of the equipment particularly exposed to abrasive wear is the transport chute and its components in the scraper conveyors made of wear-resistant martensitic steel [11,12].

The available literature on the subject contains relatively few research papers on the effect of coal and coal abrasives on the wear of steel components, especially papers clarifying the mechanisms of surface damage formation. In their research, Ngoy and Mulaba-Bafunbiandi [13] identified a linear relationship between the abrasiveness of coal and the resulting hardness of that coal. Petrica et al. [14] demonstrated declining wear and frictional force in steel samples where a hydrated mixture of coal and quartz sand was placed between them in comparison to hydrated sand alone. Labaš et al. [15] identified a significant role of the physical properties of mineral components on wear processes. 

In their paper [16], Xia et al. evaluated the impact of such factors as water content, waste rock content, coal particle size, and the Hardgrove Grindability Index (HGI) on the wear of wear-resistant steel. Tlotleng [17] investigated the effect of various factors, such as coal type, coal petrographic composition, grain composition, hard mineral content, HGI, and moisture content on coal abrasiveness against the need to ensure adequate durability of crusher and mill linings. His research showed that the content of moisture, vitrinite, and mineral matter were the key characteristics which increased the abrasiveness of coal samples. 

Wieczorek et al. [18] conducted wear tests on two wear-resistant steels in the presence of a mixture of quartz and ground coal, and of coal alone. What they managed to reveal in their study (similar to the authors of [13]) was the linear impact of the quartz content on the wear of steel surfaces. In the paper, they also proposed a wear model for surfaces having coal or coal-mineral abrasive material between them. 

Hard coals differ in regard to external petrographic characteristics. Lithotypes, i.e., petrographic varieties of hard coal, form sets characterising the structure and texture of seams, while the conventional thickness of a lithotype is 5 mm. On this basis, four basic lithotypes are distinguished, differing in lustre and colour. These are vitrinite, clarinite, durinite, and fusinite [19].

Vitrinite is characterised by a variable lustre depending on the degree of its carbonisation: at low degrees of carbonisation, the lustre is tarry, while it is glassy in coking coal, diamond-like in lean coal, and metallic in anthracite. As the carbonisation degree increases, the coal colour also changes, from tarry black to lead grey. In the seam, this lithotype usually forms streaks from 1–13 mm to 2–5 cm in thickness, as well as bands and lenses. Vitrinite crumbles easily, showing conchoidal fracture, and contains small amounts of mineral admixtures. It is characterised by the occurrence of endocleavage, i.e., a primary system of natural cracking formed by the contraction of coal gel. 

Clarinite is a heterogeneous lithotype consisting of vitrinite and durinite bands, sometimes with fusinite streaks, consequently exhibiting intermediate properties. In the seam, it forms bands or beds up to 1 m thick. In vitriniferous bands, it is usually possible to observe endocleavages whose fissures are filled with mineral admixtures. 

Fusinite is composed of fragments of charred plant parts; it is characterised by a grey-black to black colour and silky sheen and is brittle and easily abraded. It is most commonly found on the surface of the bedding of other lithotypes.

Durinite, depending on its petrographic composition, is characterised by a grey or black colour, while its lustre varies from dull to greasy-dull as the carbonisation degree progresses. According to Misiak [20], durinite characterised by the black colour is genetically related to strongly flooded peat areas, where the deposited plant material was subject to humification and gelification processes. A lighter colour of durinite, on the other hand, indicates that it formed in shallower areas of peats, where the water level was periodically lowered, leading to oxidation of the material deposited in the peat bog. Fractures in durinite are typically granular or conchoidal-granular, showing a rough surface. Durinite is hard, compact, firm, and contains no fracture fissures. Within this lithotype, one may find isolated vitrinite bands and inclusions of mineral matter, most commonly associated with the presence of clay minerals, which may be of both secondary and primary origin [21]. In the seam, durinite forms bands and beds several cm thick. 

The aim of the research addressed in this paper was to identify the properties of coal and coal-mineral abrasives associated with claystones rich in coal admixtures depending on their mineral composition. Moreover, wear tests in the presence of relatively soft abrasives were carried out and the failure mechanisms of wear-resistant steel surfaces were identified. An important novelty which the study yields is that it demonstrates the role of coal being pressed into surface irregularities, which may result in protecting them against the abrasive effect of the irregularities in the mating elements. Furthermore, what has also been demonstrated is the possibility that small fragments of hard minerals and wear products can become inactivated by the effect of the non-permanently pressed-in coal films. Not until now has the formation of coal regions on surfaces been taken into account when considering the failure mechanisms of steel surfaces in the presence of coal. In this paper, it has been highlighted that the formation of these films is induced by the softening of coal under the effect of a temperature increase caused by the friction between mating surfaces. 

## 2. Experimental Details

The research discussed in this paper comprised identification of the properties of the abrasives and steels in question, basic wear tests, and microscopic analysis of the worn surfaces. The research was divided into four stages. 

Stage 1 involved some initial procedures required to prepare the coal abrasives and claystones subject to the tests. Prior to laboratory testing, coal and claystone samples were crushed and then ground in a Retsch planetary ball mill with tungsten carbide lining. The material thus obtained was sieved through a 0.01 mm mesh screen, and the fraction below 0.01 mm was designated to be used in the laboratory analyses. Samples of the RAEX400 [22] wear-resistant steel were also prepared at this stage. The steel samples were pre-worked by turning and grinding to form rings with an internal diameter of ø45, external diameter of ø55, and thickness of 6 mm (lower sample) and 10 mm (upper sample). The surface roughness was not higher than Ra = 0.65 μm.

The nominal hardness of the surface of wear-resistant steels was 400 HB; however, the measured values ranged from 383 to 403 HB. Hardness was measured using the FM-700 hardness tester from Future-Tech (Kawasaki, Japan). The chemical composition was determined using the SPECTROMAXx spectrometer from Spectro (Kleve, Germany).

**Figure 1:**
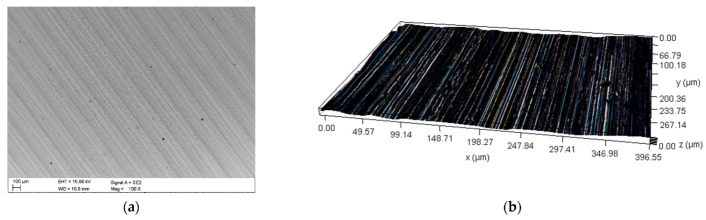
RAEX400 steel, (**a**) Surface view (SEM), (**b**) Surface profile (DM).

Figure 1a provides an image of the material surface after grinding, obtained by means of an SEM microscope. The surface profile determined using the DVM6 digital microscope from Leica (Wetzlar, Germany) is provided in Figure 1b. The surface roughness was Ra = 0.65 μm, as determined using the MahrSurf 400 profilometer from Mahr (Göttingen, Germany).

Stage 2 comprised the following identification studies of coals and claystones:

- Microscopic observations of claystones in thin plates using a polarising microscope;
- Microscopic observations using scanning electron microscopy;
- Chemical composition analysis using X-ray fluorescence (XRF);
- Phase composition analysis of coal claystones using the XRD method;
- Examination of the coals and claystones using Raman spectroscopy.

The microscopic observations were conducted within transmitted light, in thin plates, using the OPTA-TECH LAB-40 HAL diagnostic polarisation microscope featuring an image analyser from Opta-tech (Warsaw, Poland). The chemical composition analysis using X-ray fluorescence (XRF) was performed as per EN ISO 12677: 2011 using roasted samples following the determination of their ash content.

Once roasted to constant mass, the sample was melted with a commercially available mixture of lithium tetraborate, lithium metaborate, and lithium bromide (66.67%, 32.83%, and 0.5%) characterised by a flux purity adequate for XRF (from Spex, Long Island City, NY, USA). The sample to flux weight ratio was 1:9. The samples to be analysed were prepared by melting their mineralogical and grain structure until destroyed. The samples prepared in this way were measured using the PANalytical MagiX PW2424 spectrometer from PANalytical B.V. (Almedo, The Netherlands) and were calibrated using a series of certified reference materials: JRRM 121-135, JRRM 201-210, and JRRM 301-310 (Technical Association of Refractories, Tokyo, Japan). The ash content was determined according to PN-80/G-04512 by sample roasting at 815 °C to constant mass. The coal content was determined in samples ground and dried to constant mass using the SC 144 DR sulphur and carbon analyser featuring a resistance furnace from Leco Corporation (St. Joseph, MI, USA). The samples were burnt in an oxygen stream at 1350 °C. The CO_2_ content of the resulting gas was determined by measuring infrared absorption.

The phase composition tests were performed using the Panalytical X’Pert PRO MPD X-ray diffractometer from PANalytical B.V. (Almedo, The Netherlands), equipped with a cobalt anode X-ray tube (λ_Kα_ = 0.179 nm), and the PIXcel 3D detector. Diffractograms were recorded in the Bragg–Brentano geometry within the 5–100° 2Theta angle range with a step of 0.026° and a counting time per step of 80 s. The X-ray qualitative phase analysis was performed using the HighScore Plus software (v. 3.0e) and the dedicated PAN-ICSD inorganic crystal structure database.

The Raman spectroscopy analysis was performed using the Renishaw Raman Via Reflex spectrometer from Renishaw plc (Wotton-under-Edge, England), featuring the Leica Research Grade confocal microscope from Leica (Wetzlar, Germany), enabling observation of samples in reflected and transmitted light. Excitation was performed using a beam of λ = 514 nm produced by a 50 mW argon ion laser. Sample measurements were recorded over a wide wave number range from 50 to 3200 cm^−1^.

**Figure 2:**
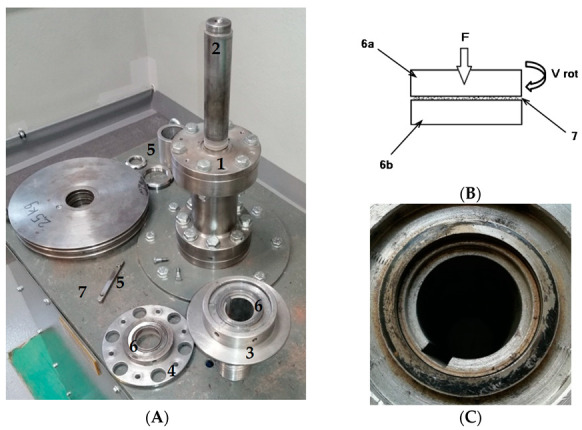
Tribotester; (**A**)—test stand, (**B**)—schematic diagram of the wear testing method, (**C**)—view of a sample immediately following the test; designations: 1—test head of the stand, 2—drive shaft, 3—upper sample holder, 4—lower sample holder, 5—fixing elements, 6—test sample (6a—upper sample and 6b—lower sample), and 7—stand base with motor.

In Stage 3, wear tests were carried out using the authors’ original ring-on-ring [23] stand (Figure 2A). The conditions simulated at the test stand corresponded to those of the actual operational wear of steel machine components in the presence of rock material. A characteristic feature of this test was that crushed grain of the abrasive material and of wear products, obtained from the damaged surface of steel samples, was constantly present between the steel samples (Figure 2B). It was assumed that after each 10 min wear cycle (Figure 2C) the samples would be cleaned, weighed, and refilled with fresh abrasive in the amount of 1 cm^3^. For each sample, the test consisted of 8 10 min cycles, and each test was repeated 3 times. The basic parameters characterising the wear test are provided in Table 1.

During the tests, the mass loss of sample u_M_ was determined according to the following Formula (1):

(1) $$u_{M}=\left(m_{\mathrm{pd}0}-m_{\mathrm{pdt}}\right)+\left(m_{\mathrm{pg}0}-m_{\mathrm{pgt}}\right)$$

where u_M_ is the sample mass loss [g], m_pd0_ is the bottom sample mass before testing [g], m_pdt_ is the bottom sample end mass [g], m_pg0_ is the upper sample mass before testing [g], and m_pgt_ is the upper sample end mass [g].

Measurement uncertainty was determined using Student’s *t*-test method for the confidence level of 0.95 and the number of tests of n = 3. The relative measurement uncertainty was less than 1% for all the cases considered. Immediately after the wear test, the temperature of the steel samples was also measured using a thermal imaging camera.

Stage 4 involved a microscopic analysis of the surface of the samples after the wear tests. The surface of the steel samples was observed using the ZEISS Supra 35 scanning electron microscope from Zeiss (Jena, Germany) using secondary electron (SE) detection at an accelerating voltage of 20 kV and a magnification of 60 ÷ 1000×. The qualitative analysis of the chemical composition in the microareas of the material subject to the tests was performed using energy dispersive spectroscopy (EDS) at an accelerating voltage of 20 kV. Prior to testing, the samples were sprayed with a thin film of gold to ensure electrical charge dissipation during the tests. At this stage, topography was studied using the Profilm3D optical interferometric profilometer from Filmetrics Inc. (San Diego, CA, USA).

## 3. Results

### 3.1. Identification Tests

**Figure 3:**
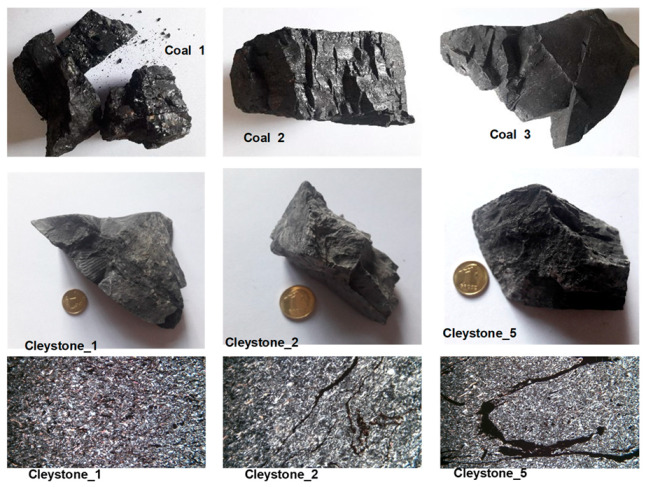
Samples (rows 1 and 2) and microstructure (row 3) of the selected coals and claystones.

The tests described in this paper were generally carried out for three coal and five claystone samples (Figure 3 shows the samples and the microstructure of the selected coals and claystones). The macroscopic observations, which were also correlated with the results of laboratory analyses, made it possible to establish that the studied coal samples belonged to three different petrographic varieties (lithotypes) of hard coal. Sample Coal_1 represented coal of glassy lustre, tarry black colour, and clearly visible endocleavage, which crumbles easily; on account of its properties, it was classified as vitrinite lithotype. The Coal_2 sample was characterised by alternating bands of glossy and dull coal, and, therefore, it was considered to be representative of the clarinite lithotype. Although the ash content (a parameter that is often reported as characteristic of a given lithotype) of samples Coal_1 and Coal_2 was slightly higher than the values commonly accepted for these varieties, the differences resided within the measurement error limits. The ash content in vitrinite typically ranges between 2 and 6%, and it is 4–12% in clarinite [24], while for the coals analysed, the amount of ash was 6.91% in vitrinite (Coal_1) and 15.85% in clarinite (Coal_2).

The Coal_3 sample showed a dull lustre and grey colour, and it was characterised by a rather significant compactness and hardness. Based on the macroscopic features, this sample was identified as durinite. Due to the high ash content, reaching 35.90%, this sample could be considered as representative of high ash coals, with the ash content up to 40%. Gabzdyl [24] defines coals containing 30–50% of ash as very low purity coals.

In macroscopic terms, claystones exhibit similar characteristics, such as grey to dark grey colour associated with the presence of dispersed coal matter in their composition. The structure of claystones is pelitic and their texture is compact. When the rock comes into contact with water, a distinctive smell of mortar is released, which implies the presence of clay minerals in their composition.

Microscopic images show clay minerals to form colourless microcrystalline grains, showing some grey first-order interference colours in parallel polarisers. Quartz occurs as generally allomorphic colourless specimens, accompanied by sharp-edged shards; and similar to clay minerals, it is characterised by first-order interference colours. Muscovite forms elongated, autimorphic grains, with third-order interference colours. In certain samples of claystone, one can observe a peculiar form of muscovite, where elongated grains are arranged perpendicularly to the direction of pressure, providing the rock texture with directional characteristics. All the studied claystones contained coal matter; however, it occurred in quite diversified forms. Besides the dispersed pigment, one could observe coal slivers of various sizes and elongated forms of laminae, which were also accountable for the directional nature of the texture of the claystones.

**Figure 4:**
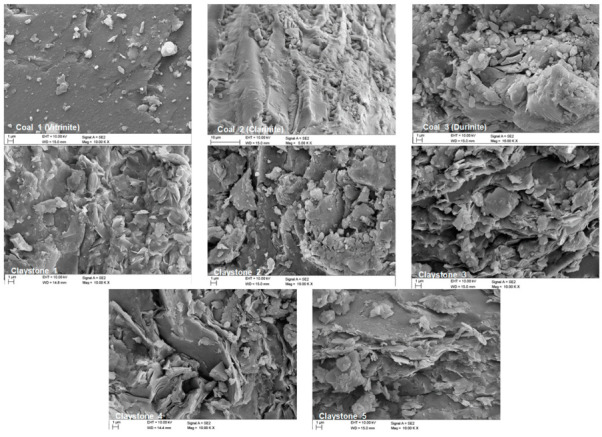
Example of micrographs of claystones and coals, SEM.

Figure 4 provides microscopic images of the coals and claystones in question, obtained by scanning electron microscopy. The results of the analyses of chemical composition, as well as of the ash and carbon (C) content in the test samples, are summarised in Table 2.

What proved to be dominant in terms of the quantitative share in all the analysed coal samples was the loss on ignition (LOI), i.e., the loss caused by roasting, including the organic matter decomposed at temperatures below 600 °C, crystallisation water, nitrites, boron salts, etc. In Coal_1, the LOI reached the highest value of 93.09%; in Coal_2, the LOI content dropped to 84.15%, while in Coal_3, the LOI was quantitatively lowest, as it came to 64.10%.

The mineral substance present in the coals studied is identified primarily by its silica content, with the highest amount of SiO_2_ found in sample Coal_3 (18.52%) and the lowest in sample Coal_1 (2.70%). In sample Coal_2, the SiO_2_ content reached 7.71%. Apart from silica, the chemical composition of the coals included Al_2_O_3_ (1.83% in sample Coal_1, 3.19% in sample Coal_2, and 6.91% in sample Coal_3) and Fe_2_O_3_ (0.69% in sample Coal_1, 1.91% in sample Coal_2, and 6.63% in sample Coal_3). Other elements were present in smaller amounts, their content reaching 1%. The results of the chemical analysis correlate with the ash content determined for individual samples. Ash is a solid residue left after coal combustion; its composition and content are not related to the carbonisation process, but mainly to the sedimentation conditions and the type of vegetation. For sample Coal_1, the ash content was the lowest at 6.91%; in sample Coal_2, it was 15.85%, while the highest value of 35.90% was established for sample Coal_3. The content of elemental carbon in coal was the highest in sample Coal_1 (75.2%); in sample Coal_2, it reached 69.31%, and it was the lowest in sample Coal_3, being 52.11%. The dominant component in the chemical composition of the claystones was silica (SiO_2_). Its content varied between 58.80% and 64.39%. The second most abundant compound in all the studied samples was Al_2_O_3_, its content ranging from 27.90% to 29.05%. The presence of silica and alumina was mainly connected with the presence of silicate minerals: quartz, kaolinite, and muscovite.

In claystones, the following fractions were observed: K_2_O (2.95–3.99%), Fe_2_O_3_ (0.98–3.85%), TiO_2_ (1.25–1.39%), and MgO (0.56–1.51%), associated with the presence of aluminosilicates. Other compounds were found to occur in smaller quantities, their content generally not exceeding 1%.

**Figure 5:**
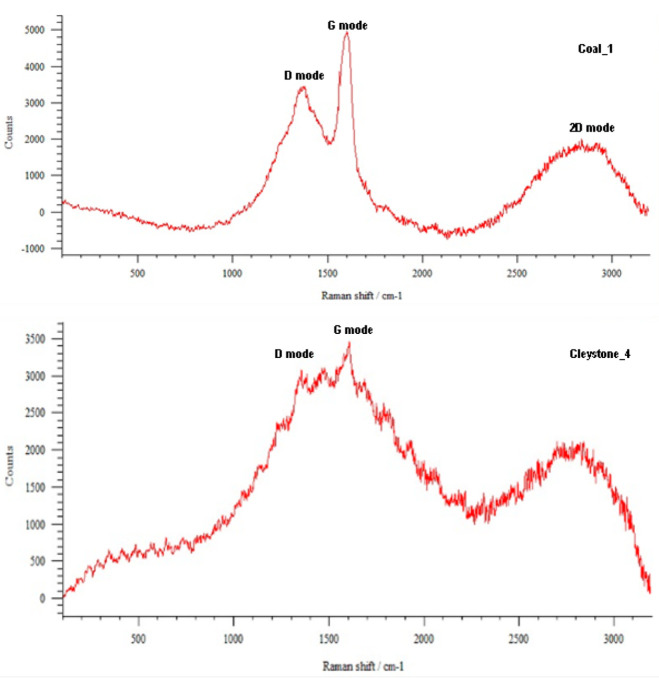
Results of the Raman spectroscopic analysis of selected samples.

The results of the examinations performed using the Raman spectroscopy of the selected abrasives are presented in Figure 5.

**Figure 6:**
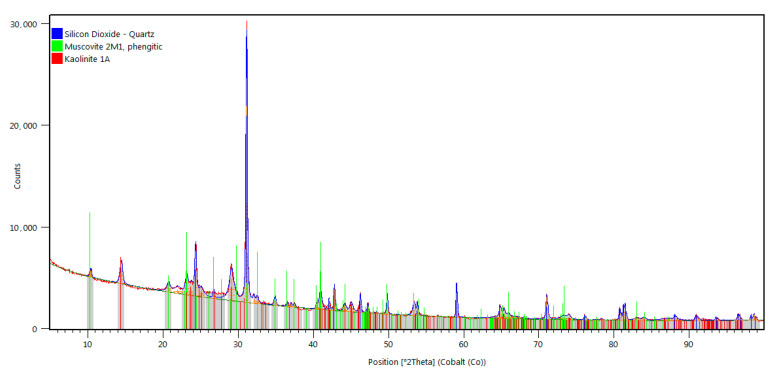
Sample claystone diffractogram.

X-ray phase identification tests revealed that the clay mineral group was represented by kaolinite (Al_4_[Si_4_O_10_](OH)_8_). The diffractograms of all the claystones studied contained distinctive peaks of quartz (SiO_2_) and muscovite (KAl_2_(OH,F)_2_AlSi_3_O_10_), observed in the course of the microscopic observations in thin plates (Figure 6). Diffraction lines running from quartz (SiO_2_), having a hexagonal crystal lattice (ISCD: 98-016-8354), and from muscovite (KAl_2_(OH,F)_2_AlSi_3_O_10_), having a monoclinic crystal structure (ICSD: 98-007-7497), were also identified on the diffractograms of all the claystones studied.

### 3.2. Wear Tests

**Figure 7:**
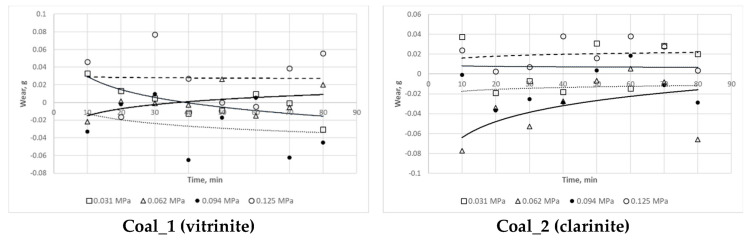

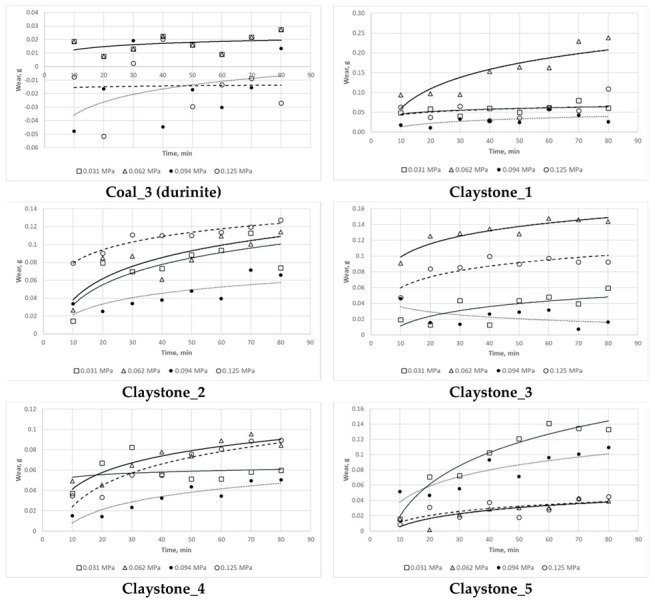
Wear test results for the coal and claystone abrasives under consideration.

The results of the wear tests are summarised in Figure 7. They imply that there is no clear relationship between the mass loss in the steel samples and the type of the abrasive material used, based on a specific petrographic variety of coal. It was only noticed that, when the Coal_1 (vitrinite) and Coal_2 (clarinite) abrasive was used, the highest mass loss in the steel samples occurred at a stress of 0.125 MPa, while for the abrasive based on Coal_3 (durinite), the highest mass loss in the steel samples occurred at a stress of 0.062 MPa.

However, it can be generally concluded that the petrographic variety of coal had no effect on the wear tests. Having analysed the results obtained for the mass loss in a function of time at a given stress, one could establish no distinctive change trend for the coal samples subject to testing. In fact, an increase in mass was observed in some cases following the wear cycle. No clear trend could be identified for individual samples in a function of load either, making it impossible to rank the respective lithotypes in terms of their impact on abrasive wear.

In the case of samples Claystone_1, Claystone_3, and Claystone_4, the highest values of mass loss were recorded at a stress of 0.062 MPa, while for sample Claystone_2 the highest values were recorded at a stress of 0.125 MPa, and for sample Claystone_5 the highest values were recorded at a stress of 0.031 MPa. With regard to the lowest mass losses, these values were observed to be obtained for a compressive stress of 0.094 MPa for all the samples except Claystone_5.

The lack of a clear relationship between load and mass loss indicates that, in the case of the coal and claystone abrasives taken into consideration, there were some conditions which disturbed the typical relationship observed for the abrasives, namely that wear increased as load increased. This problem is explained in Section 3.3 and Section 4 along with the observed increase in the sample mass. On account of the ambiguous effect of load on wear, the determination of the Archard coefficient was disregarded in this study.

Immediately after the tribotester was stopped, the surface temperature of the samples was measured. The measurements indicated moderate surface temperatures, not exceeding 50 °C. The relatively low values of the temperatures measured were most probably attributable to the heat flow from the steel samples towards the inside of the stand as a consequence of the thermal conduction phenomenon. Instantaneous temperature values at the contact point between the mating surfaces were higher, which implies that the abrasives may have been subject to higher thermal loads than those recorded with the camera.

### 3.3. Surface Analysis Following Wear Tests

**Figure 8:**
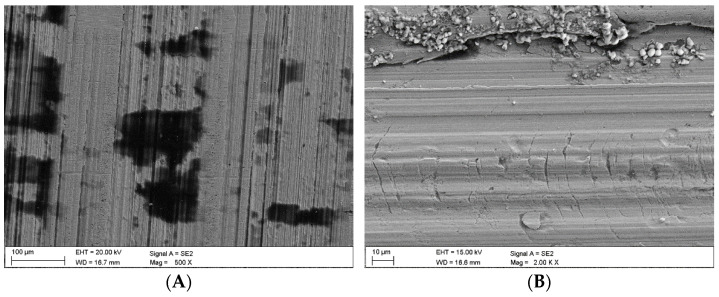
Coal_1; (**A**)—coal abrasive material pressed into the irregularities caused by the grinding wheel action, (**B**)—delamination damage and traces of fatigue cracks.

**Figure 9:**
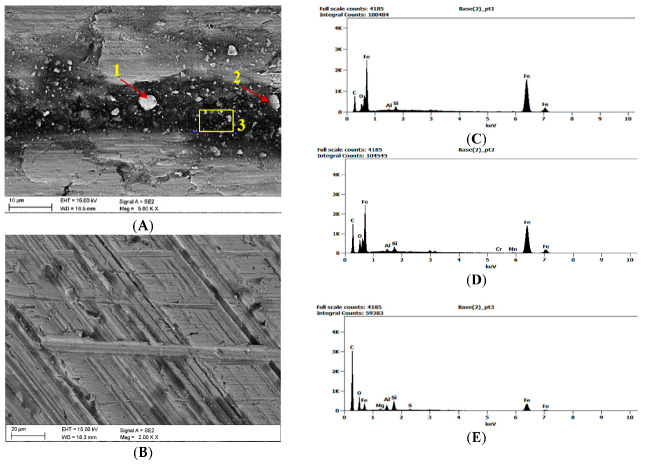
Image of the surface of the steel samples worn in the presence of the Coal_2 abrasive; (**A**)—abrasive coal film on the steel sample surface along with the inactivated wear products, (**B**)—microscratches against the background of grinding traces and coal films, (**C**–**E**)—EDS spectra determined at points 1, 2, and in area 3.

**Figure 10:**
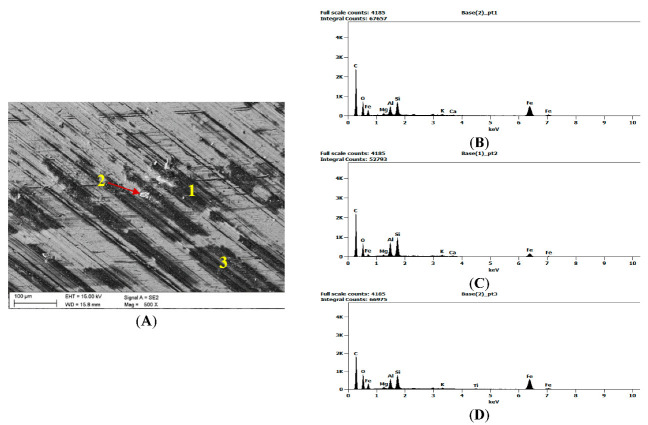
Image of the surface of the steel samples worn in the presence of the Coal_3 abrasive; (**A**)—coal abrasive layer on the steel sample surface, (**B**–**D**)—EDS spectra determined at points 1, 2, and 3.

Once the wear tests were completed and the steel samples cleaned, the latter were examined using a scanning electron microscope to evaluate the impact of the coal and claystone abrasives. In all the coal samples, one could notice rather numerous fragments of the pressed-in films of this material. Coal was particularly observed to have penetrated the areas of the irregularities which developed at the stage of the sample surface grinding. This is clearly visible in Figure 8A for the Coal_1 abrasive sample. Figure 8B shows extensive delamination of the steel layer and initial stages of fatigue cracks. Similar coal films pressed into the surface of the samples are visible in abrasive material samples Coal_2 (Figure 9) and Coal_3 (Figure 10).

**Figure 11:**
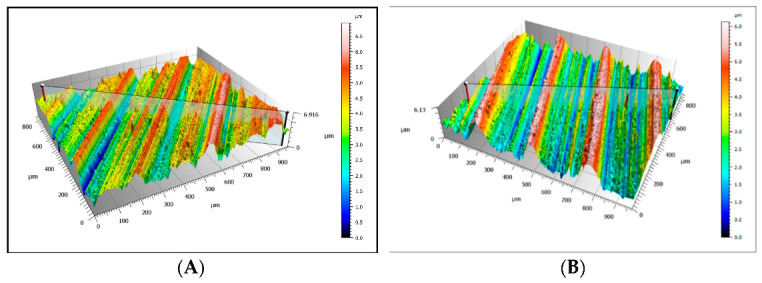
Surface profilograms of the steel samples worn in the presence of the Coal_1 abrasive; (**A**)—determined for a load of 0.062 MPa, (**B**)—determined for a load of 0.094 MPa.

However, in the case of the Coal_1 and Coal_2 abrasives, microscopic observations also revealed the presence of crushed wear products originating from the surface of the steel samples. This is confirmed by the EDS spectra shown in Figure 9A,B and Figure 10A,C. This demonstrates the ability of the compressed layer to capture wear products and possibly also some hard minerals, such as SiO_2_ and Al_2_O_3_. Visible traces of processing by grinding are characteristic of steel samples worn in the presence of coal. This is particularly evident in the profilograms shown in Figure 11, determined at various loads. These profilograms differ in terms of the depth and width of indentations, which resulted from the intensity of the abrasive action on the surfaces.

**Figure 12:**
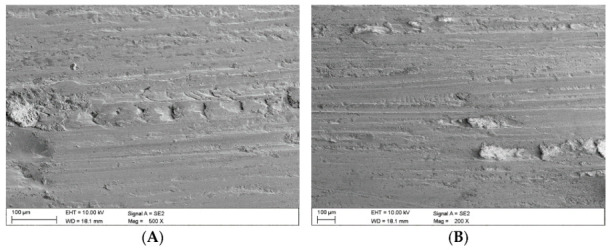
Images of the surface of the steel samples worn in the presence of the Claystone_1 abrasive; (**A**)—scaly surface cracks, (**B**)—microscratches filled with fragmented claystone grains.

**Figure 13:**
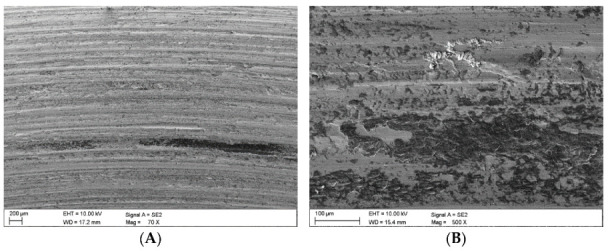
Images of the surface of the steel samples worn in the presence of the Claystone_2 abrasive; (**A**,**B**)—microcracks filled with coal matter contained in the claystones.

**Figure 14:**
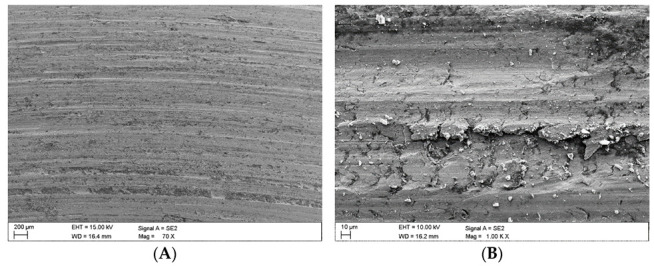
Images of the surface of the steel samples worn in the presence of the Claystone_3 abrasive; (**A**)—microscratches filled with crushed claystone grains, (**B**)—scaly surface cracks against the background of the microscratches.

**Figure 15:**
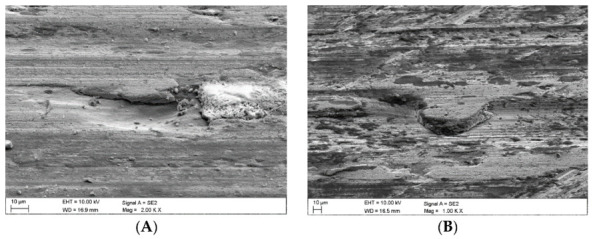
Images of the surface of the steel samples worn in the presence of the Claystone_4 abrasive; (**A**,**B**)—flat surface chipping.

**Figure 16:**
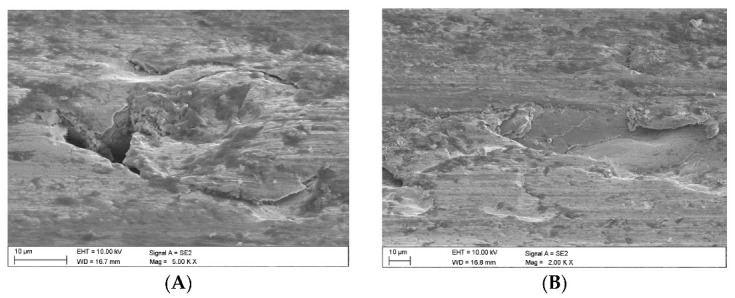
Images of the surface of the steel samples worn in the presence of the Claystone_5 abrasive; (**A**)—surface cracks penetrating deep into the surface layer, (**B**)—flat surface chipping.

In the case of the claystone-based abrasives, various forms of damage to the surface of the samples were observed:

- Microscratches on the surface of the test samples, partially filled with crushed claystone grains (Figure 12B and Figure 14A) or coal matter (Figure 13A,B) contained in them;
- Scaly surface cracks (Figure 12A and Figure 14B) initiating surface delamination damage;
- Flat fatigue-induced chipping of the steel surface (Figure 15A,B and Figure 16B);
- Surface cracks penetrating the superficial layer of the steel samples (Figure 16A).

What can be noticed in the subsequent images is that different forms of damage may be adjacent to one another (Figure 14B), while traces of previous machining are not visible.

## 4. Discussion

With reference to the wear test results and observations provided in Section 3, no relationship between the parameters characterising wear and load could be established. In the case of coal, one could even establish the kind of pressing-in mechanism which pertained to the surface irregularities formed at the stage of sample production and to the microcracks caused by the crushed wear products.

The coal pressing-in phenomenon is connected with its plastic properties, which manifest themselves under the impact of the increased temperature, caused by friction processes, on the abrasive. Hard coal chippings are characterised by low hardness, which is why the skid between them causes them to become pressed down into the scratches and fissures present on the surface of the steel sample. A bulge is usually formed where the coal has been pressed in, which may grow as a result of further abrasive loading and cause plastic deformation. The next stage is the formation of cracks [16].

This behaviour of coal abrasive is typical of polydisperse systems, which manifests itself in a number of ways, including through swelling capacity, plastic deformation under the impact of forces acting tangentially to the surface, and transition to the plastic state due to temperature effects. In terms of colloid chemistry, the plastic state of coals is a system composed of a dispersing medium and a dispersed medium. It has been demonstrated that hard coals decompose when heated, transforming into the softening state at first, then into the plastic state, only to solidify, thus forming a product with an altered structure, devoid of gaseous and liquid components. A semi-coke or coke is formed, depending on the thermal conditions applied.

As a result of the friction of the mating surfaces, there was a noticeable increase in the temperature of the abrasive material between them. This could have favoured the densification of the abrasive grains and their pressing into the surface irregularities [25,26]. According to Berkowitz’s theory [27,28], the transition to a plastic state is conditioned by the formation of a film on the surface of coal micelles, which is built up by some of the components of the coal matter. The formation of this film, which acts in a similar way to a solid lubricant, and the thermal weakening of the bonds between the micelles allow the coal micelles to move in relation to each other. 

As already mentioned, following the wear tests in the presence of coal, one could notice primary machining marks on the steel samples (see Figure 3). It was in these cracks that pressed-in coal could be found, but there was also a coal film formed, partially protecting the surface from the cutting action of the wear products formed mainly as a result of surface fatigue chipping. An interesting observation of the worn surface was the presence of its metallic fragments in the non-durable coal film. These observations indicate the positive functions of the pressed-in coal:

- Protection from the destructive action of the peaks of the irregularities of the mating surfaces;
- Inactivation of the fragmented pieces of hard abrasive materials.

The formation of discontinuous and only partially compacted coal film causes variable loads to be transferred to the superficial layer of steels, resulting in fatigue chipping that can produce further wear products. Nevertheless, it should also be emphasised that microscopic analysis revealed only a few such defects (however, they were typical of claystones).

**Figure 17:**
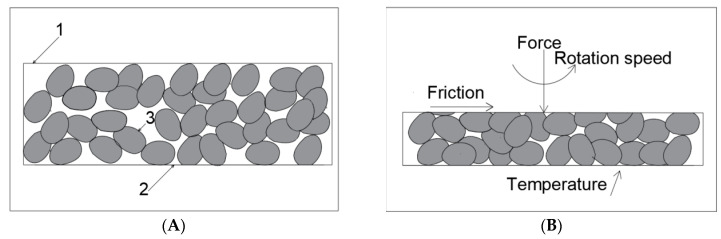

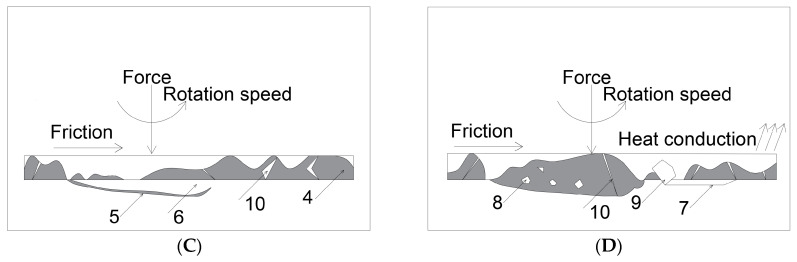
Model for the formation of unstable coal films; (**A**)—preliminary phase, (**B**)—initial compaction phase, (**C**)—compaction and surface protection phase, (**D**)—phase of chipping and inactivation of wear products; designations: 1—upper sample, 2—lower sample, 3—coal grains, 4—compacted coal film, 5—fissure filled with coal, 6—flat fatigue chipping, 7—microcut, 8—fragmented chippings in the coal film, 9—incompletely fragmented chipping particle, and 10—discontinuity of the layer.

Figure 17 presents a model for the formation of this type of unstable coal film. This model predicts four phases:

- Preliminary phase with uncompacted abrasive (Figure 17A);
- Initial phase of frictional compaction of the coal abrasive under load and increasing node temperature due to frictional forces (Figure 17B);
- Formation of a compacted coal film; in this phase, fatigue cracks may develop on the surface (Figure 17C);
- Inactivation of hard abrasive fragments by the discontinuous coal film (Figure 17D).

A local increase in the coal abrasive temperature during wear tests may also lead to an increase in the content of element C in the coal. This was demonstrated in a previous study [29], where an increase of more than 1% in the content of element C was observed in the coal abrasive compared to its amount in the initial sample. This implies that a sudden increase in temperature can affect the transformations of the abrasive material components.

Coal, as it has been demonstrated, can be relatively non-invasive in wear processes on account of its low abrasiveness and capacity to form separating layers on mating surfaces without causing significant damage to steel surfaces. However, it should be remembered that a pure coal abrasive is rarely available under the operating conditions of mining machinery; usually, it is a mixture of coal and waste rock. Moreover, coal may contain mineral admixtures that have entered the sediment during the formation process in the sedimentation basin [13,30,31]. Such an abrasive medium is non-uniform, often containing fractions of quartz, pyrite, and some content of coal which can also be mixed with water, consequently forming an abrasive slurry mixture [14].

The claystones used during this study contained quite significant amounts of coal matter, which was present in a dispersed form (as pigment), explaining the dark grey colour of the rock. It was established that, in the presence of fragmented claystone grains, wear was not intensive, which should mainly be attributed to the plastic properties of the clay minerals, enabling the phenomenon of partial pressing into the primary cracks formed at the stage of sample production as a result of grinding, and into the secondary cracks, i.e., those caused by fatigue chipping, and the cuts caused by hard aggregates of the abrasive material and chipped steel (wear products). Observations using scanning electron microscopy indicated traces of microscratching, which were caused by the impact of the wear products on the surface of the steel samples. Wear products are understood to be small steel particles detached from the top layer of the steel sample. Their formation is connected with the cracking of the superficial layer under the impact of high pressure and microfatigue, which leads to the formation of wear products, which behaved similar to the hard mineral abrasive during the tests performed.

As a result of the continuous interaction between wear products and hard abrasive grains on the superficial layer of the steel sample, one could observe further delamination and enlargement of the scratches previously formed [32,33]. The final effect of the fatigue process was the separation of a fragment of the superficial layer in the form of a characteristic thin scale.

However, compared to the results of the tests conducted in the presence of coal alone, the diagrams provided in Figure 10 show that wear processes took place despite the fact that the abrasive material was mainly a mixture of soft matter composed of clay mineral grains and coal. However, in the case of claystone, another important component was determined in their composition, which could not have been recognised macroscopically. During the microscopic observations in thin plates, fine quartz grains were identified. The chemical composition analysis showed that the claystones could contain up to 64.39% of SiO_2_ (sample Claystone_2), which should be interpreted as symptomatic of the presence of quartz. The results of mineralogical and chemical tests correlate with the results of the wear tests. The effect of quartz cannot be excluded in the process of microcracking (the chemical analysis indicated quite a significant share of silica in the chemical composition of the coals tested). Quartz actually plays a significant role in the composition of the mineral matter present in coal, predominantly clastic quartz, which is deposited by wind or water.

## 5. Conclusions

Based on the test results, the following detailed conclusions have been formulated:

1. The wear tests conducted on the coals in question revealed low values of mass loss, while following certain wear cycles a mass gain was observed.
2. No effect of the load attributable to coal type I (lithotype) on the abrasive wear was observed during the tests.
3. At the end of the tests, the pressed-in coal grains were found on the tested surfaces of wear-resistant steel. The pressed-in coal also formed films on the surfaces of the samples.
4. The coal films thus formed exhibited properties enabling them to provide protection against the destructive effect of the peaks of the irregularities in the mating surfaces and to inactivate the fragmented chippings of hard abrasives. The formation of the non-durable coal films was facilitated by the increase in the temperature of the test node due to friction; the temperature increase affected the plasticisation of the coal abrasive material subject to testing. The authors of this paper have proposed a model which explains the formation of the said coal films as well as the mechanism behind this process.
5. The main steel surface failure mechanisms acting in the presence of the coal were fatigue chipping and abrasion due to wear products, while in the case of the claystones, these were microcutting, formation of scaly surface cracks, delamination, and deep cracking.
6. With regard to the claystones, grains of the abrasive material were found to have been pressed into the surface irregularities, but they did not create any layered superficial forms.

## Figures and Tables

**Table 1:** Main parameters of the wear tests.

Parameter	Value
Contact surface area S, mm^2^	785.3
Pressing force F, N	147.2
Mass of the set load, kg	15.0
Compressing stress σ, MPa	0.187
Rotational speed of the moving sample, RPM	149.1
Average linear speed of the moving sample, m/s	0.29
Tests duration, min	8 × 10
Sliding distance, m	1390
Number of test repetitions for each variant	3

**Table 2:** Results of the chemical composition analysis of the coals and claystones studied.

	Coal_1	Coal_2	Coal_3	Claystone_1	Claystone_2	Claystone_3	Claystone_4	Claystone_5
Al_2_O_3_, %	1.83 ± 0.18	3.19 ± 0.16	6.91 ± 0.35	27.90 ± 1.35	28.07 ± 0.95	29.05 ± 1.11	27.99 ± 1.14	28.21 ± 1.28
BaO, %	-	-	-	0.07 ± 0.005	0.20 ± 0.02	0.06 ± 0.005	-	-
C, %	75.2 ± 3.01	69.31 ± 2.77	52.11 ± 2.08	n.i.	n.i.	n.i.	n.i.	n.i.
CaO, %	0.45 ± 0.22	0.84 ± 0.42	0.80 ± 0.40	0.18 ± 0.02	0.05 ± 0.005	0.12 ± 0.01	0.06 ± 0.005	0.06 ± 0.005
Cl, %	-	-	-	0.62 ± 0.07	0.11 ± 0.01	0.03 ± 0.001	-	-
Cr_2_O_3_, %	<0.01 ± 0.001	0.01 ± 0.001	0.02 ± 0.001	0.03 ± 0.004	0.03 ± 0.004	0.03 ± 0.004	-	-
CuO, %	-	-	-	0.01 ± 0.001	0.006 ± 0.001	0.004 ± 0.001	-	-
Fe_2_O_3_, %	0.69 ± 0.34	1.91 ± 0.19	6.63 ± 0.33	3.85 ± 0.30	2.05 ± 0.28	0.98 ± 0.11	3.35 ± 0.32	3.01 ± 0.31
Ga_2_O_3_, %			-	0.005 ± 0.001	0.005 ± 0.001	0.006 ± 0.001	-	-
K_2_O, %	0.13 ± 0.07	0.43 ± 0.22	0.96 ± 0.48	3.99 ± 0.45	3.78 ± 0.42	2.95 ± 0.33	5.64 ± 0.01	3.14 ± 0.01
MgO, %	0.25 ± 0.12	0.64 ± 0.32	0.90 ± 0.45	1.51 ± 0.11	0.99 ± 0.09	0.56 ± 0.05	1.27 ± 0.11	1.23 ± 0.11
MnO, %	0.01 ± 0.001	0.04 ± 0.002	0.26 ± 0.13	0.04 ± 0.01	0.02 ± 0.005	0.01 ± 0.001	0.04 ± 0.005	0.03 ± 0.005
Na_2_O, %	0.04 ± 0.02	0.08 ± 0.04	0.09 ± 0.04	0.74 ± 0.08	0.49 ± 0.05	0.26 ± 0.04	0.6 ± 0.07	0.39 ± 0.05
NiO, %	-	-	-	0.01 ± 0.001	0.008 ± 0.001	0.01 ± 0.001	-	-
P_2_O_5_, %	0.18 ± 0.09	0.24 ± 0.12	0.12 ± 0.06	0.13 ± 0.02	0.05 ± 0.001	0.07 ± 0.01	0.09 ± 0.01	0.05 ± 0.01
PbO, %	-	-	-	0.006 ± 0.001	0.005 ± 0.001	-	-	-
Rb_2_O, %	-	-	-	0.02 ± 0.005	0.02 ± 0.001	0.02 ± 0.002	-	-
SiO_2_, %	2.70 ± 0.13	7.71 ± 0.39	18.52 ± 0.93	58.80 ± 2.11	62.47 ± 1.98	64.39 ±2.54	59.77 ± 3.04	62.69 ± 2.99
SO_3_, %	-	-	-	0.66 ± 0.08	0.18 ± 0.01	0.15 ± 0.01	-	-
SrO, %	-	-	-	0.01 ± 0.001	0.01 ± 0.001	0.008 ± 0.001	-	-
TiO_2_, %	0.06 ± 0.03	0.15 ± 0.07	0.32 ± 0.16	1.30 ± 0.11	1.39 ± 0.10	1.25 ± 0.12	1.19 ± 0.12	1.19 ± 0.11
LOI, %	93.09 ± 9.31	84.15 ± 8.42	64.10 ± 6.41	-	-	-	-	-
Ash, %	6.91 ± 0.69	15.85 ± 1.58	35.90 ± 3.59	-	-	-	-	-

## Data Availability

Not applicable.
